# A comparison between the planned and actual external loads experienced during training in a professional, male basketball team

**DOI:** 10.3389/fpsyg.2026.1768705

**Published:** 2026-04-07

**Authors:** Aaron T. Scanlan, Cecilia Smith, Stephanie Shirley, Rogan Bartlett, Nathan Elsworthy, Shaoling Zhang, Davide Ferioli, Sergio José Ibáñez, Joshua H. Guy

**Affiliations:** 1 School of Health, Medical and Applied Sciences, Central Queensland University, Rockhampton, QLD, Australia; 2 S.P.O.R.T. Research Cluster, Central Queensland University, Rockhampton, QLD, Australia; 3 Cairns Taipans Basketball Club, Cairns, QLD, Australia; 4 Division of Sports Science and Physical Education, Tsinghua University, Beijing, China; 5 Department of Biomedical Sciences, Dental Sciences and Morpho-Functional Imaging, University of Messina, Messina, Italy; 6 Training and Sports Performance Optimization Group, Faculty of Sports Sciences, Department of Didactics of Music, Plastic and Body Expression, University of Extremadura, Cáceres, Spain

**Keywords:** athlete monitoring, microsensor, NBL, PlayerLoad, prescription, training load

## Abstract

**Introduction:**

Comparisons between the training loads encountered by players to those planned by coaches using external load metrics are largely absent in the team sport literature. Consequently, this exploratory, observational study compared the planned and actual external loads during training sessions in a professional, male basketball team, overall and separately according to key factors.

**Methods:**

The planned load was determined by an experienced head coach, while the actual load was measured in 23 players during 138 training sessions across two seasons using microsensor technology. Planned and actual load data were determined using key variables including accumulated PlayerLoad^TM^ (PL) in arbitrary units (AU), relative PL (AU·min^−1^), and duration (min). Sessions were categorized according to key factors including season (2022–2023 and 2023–2024), intended difficulty (low: PL = < 375 AU; medium: PL = 375–475 AU; and high: PL = >475 AU), number of days before a game that training was conducted (1 day before games, 2 days before games, and three or more days before games), season phase (pre-season and in-season), and venue of the upcoming game (home and away). Paired *t*-tests, Cohen's *d*_*z*_ effect sizes, and Bland-Altman analyses were used to compare planned and actual external loads overall across all sessions combined and for each category within each factor.

**Results:**

A lower accumulated PL was encountered in sessions compared to that planned (401 ± 81 AU vs. 422 ± 72 AU, *p* < 0.001, *small* effect). This effect was predominantly attributed to a reduced session intensity being encountered compared to that planned (5.97 ± 0.91 AU·min^−1^ vs. 6.13 ± 0.84 AU·min^−1^, *p* = 0.02, *small* effect) given session duration was relatively consistent (67.7 ± 11.4 min vs. 69.2 ± 9.5 min, *p* > 0.05, *trivial* effect). Considering key factors, a reduced accumulated PL encountered in training compared to that planned was most strongly apparent in the second season monitored, in sessions planned to elicit high loads, and in sessions conducted on the day before games (*p* < 0.001, *moderate* effects).

**Discussion:**

Our findings highlight the extent of misalignment between planned and actual external training loads within different contexts for a specific approach adopted in the monitored team. Accordingly, end-users can interpret this level of agreement in line with their own standards, and utilize the recommendations we provide to strengthen this agreement further.

## 1 Introduction

Athlete monitoring systems are commonplace in competitive basketball teams, with “training load” being the most popular construct measured within these systems (Burger et al., 2024). Load data represents what athletes do (i.e., external load) and how they respond (i.e., internal load) during training sessions and competitive games (Jeffries et al., 2022). External load data are particularly useful for basketball coaching staff in training scenarios, reflecting the physical stimuli encountered by their players during prescribed drills (West et al., 2021). In other words, there is a direct link between the contents of training sessions planned by coaching staff and the physical demands undertaken by their players during those sessions. The external loads experienced will also strongly contribute to the psychological and physiological adaptations, as well as performance outcomes, that ensue among players (Jeffries et al., 2022). It is therefore crucial that the external loads experienced by basketball players during training sessions align with the planned load informed by the expertise of coaches.

With access to suitable data, basketball coaches may plan precise external loading schemes for players during training sessions by manipulating specific drill types, durations, and intensities. It has been shown that basketball coaches can anticipate the typical external loads to be experienced during specific training drills with relatively low error based on data derived from only a few sessions (O'Grady et al., 2020a). However, it is unclear whether the planned external loads prescribed during training sessions translate to what is actually experienced by players. For instance, coaches may not accurately anticipate the physical demands involved across all prescribed drills or make *ad hoc* adjustments in real-time during training sessions based on their perception of team effort and performance. Indeed, real-time changes based on observation, rather than using training load data, are common in basketball settings (Fox et al., 2020). Moreover, delivery of prescribed external loads can be challenging in group-based team environments due to individual differences among players (Staunton et al., 2020). In this way, if there is a misalignment between the planned and actual external loads in basketball teams, insufficient or excessive loading stimuli may be applied to players with potential to negatively impact their health, susceptibility to injury, and performance (Chan et al., 2024; Gabbett, 2020).

Although external load data and monitoring procedures for basketball players have been readily investigated–evidenced by several reviews in the area (O'Grady et al., 2020b; Tuttle et al., 2024; Fox et al., 2017; Russell et al., 2021; Espasa-Labrador et al., 2023; Martinho et al., 2025; Power et al., 2022)–no research has performed a fundamental analysis into whether the predetermined external load prescribed by coaching staff is actually experienced during training in basketball settings. Instead, existing basketball research related to this topic has been confined to exploring prescribed training loads from coaches using rating of perceived exertion (RPE) metrics (Kraft et al., 2020; Staunton et al., 2020). More precisely, the head coach of a professional, female basketball team was shown to underestimate the intensity of moderate-to-hard training drills (planned as an RPE = 3–5 on a 10-point scale), which yielded comparable (*p* > 0.05) external load intensities (indicated by average net force) performed by their players compared to drills planned as hard-to-very hard (RPE = >5–7), and very hard-to-maximal (RPE = >7–10; Staunton et al., 2020). In contrast, head coaches of male and female, collegiate basketball teams were shown to plan higher (*p* < 0.05) session intensities (RPE) and comparable (*p* > 0.05) session volumes (session-RPE-load, determined as RPE ^*^ session duration) to those experienced by their players (Kraft et al., 2020). While providing useful insight, the RPE variables explored in these studies may hold limited prescriptive utility given they are internal load indicators influenced by several factors other than the external load experienced, like age, body composition, physical fitness, health status, and psychosocial aspects (Brink et al., 2014; Impellizzeri et al., 2019), with coaches highlighting their lack of confidence in predicting athlete responses to training (Anyadike-Danes et al., 2025). Moreover, these previous basketball studies were conducted across acute timeframes (~1–6 weeks) in the pre-season (Staunton et al., 2020) or early competitive season phase (Kraft et al., 2020). Consequently, longitudinal research is needed comparing the planned training loads to those encountered using external load variables with strong prescriptive appeal across a season in basketball settings.

In performing such analyses, it is likely important to consider key factors that could impact the planned and actual external loads among basketball players, such as the intended session difficulty, proximity to games, season phase, and game venue. In this way, recent systematic evidence indicates mismatches between planned and actual RPE-based training loads tend to emerge when considering sessions categorized according to their intended difficulty (e.g., low, moderate, and hard) across various team sports (Paul et al., 2021). Likewise, training objectives and/or availability to train–and consequently the external loads–vary depending on the proximity of the training session to games (Wellm et al., 2023), season phase in which training is taking place (Williams et al., 2022), and location of games (home vs. away; Staunton et al., 2018). Therefore, this exploratory study aimed to: (1) longitudinally compare the external load planned by the head coach to the actual external load experienced by players during training sessions in a professional, male basketball team; and (2) stratify these comparisons according to the season, intended session difficulty, proximity to games, season phase, and game venue.

## 2 Materials and methods

### 2.1 Design

An exploratory study involving a retrospective observational design was adopted to quantify and compare the external load planned by the head coach and the actual external load experienced by professional, male basketball players during training sessions. The Strengthening the Reporting of Observational Studies in Epidemiology (STROBE) statement was followed in preparing this manuscript (von Elm et al., 2008).

### 2.2 Participants

This study involved 23 adult male, professional basketball players (age: 25.0 ± 3.1 years; height: 197.3 ± 7.5 cm; body mass: 92.9 ± 7.8 kg) and the head coach from the same team competing in the Australian National Basketball League (NBL). The head coach had over 15 years of coaching experience, including 5 years of coaching in national-level competitions and 1 year of international coaching representation. The NBL is a professional competition comprising 10 teams across Australia and New Zealand, and is considered among the top basketball leagues globally. The NBL is considered a Tier 4, elite-level competition in the Participant Classification Framework (McKay et al., 2021). Players were monitored during all on-court team training sessions across two full seasons (2022–2023 and 2023–2024 seasons). Across the seasons, the pre-season phase lasted ~7 weeks, the in-season phase lasted 18–20 weeks, and the post-season phase (including play-in tournament) lasted ~2 weeks. Throughout each in-season phase, two ~1–2-week league-wide breaks from competition were administered to accommodate players competing in international competitions. Moreover, the monitored team participated in a series of exhibition games against teams in the National Basketball Association (NBA) across North America during a ~3-week period early in the 2023–2024 season. Any data collected during these periods (*n* = 9 sessions) were not considered in analyses given the irregularity in training environment regarding the organization, scheduling, and team participation among sessions.

Monitored sessions predominantly consisted of shooting drills, conditioning work, and game-based drills prescribed by coaching staff. To be eligible for inclusion in the study, players had to be a registered player with the team and participate in on-court team training sessions throughout the monitoring period (one or both seasons). Across the two seasons, 8 players competed only in the 2022–2023 season, 10 players competed only in the 2023–2024 season, and 5 players competed in both seasons. There was no criterion set according to the minimum number of sessions in which players had to complete for inclusion–although, for data from individual sessions to be included, players had to sufficiently partake in prescribed drills without any individualized modifications (e.g., rehabilitative return-to-play plans). In this regard, individual player data were excluded within specific sessions when they: (1) did not participate adequately across all drills within the session due to excessive non-involvement and/or substitutions (set at < 80% of the session duration administered to represent participation in a substantial portion of the session); (2) completed a modified training session; or (3) had their device malfunction. Data for entire sessions were also excluded when < 5 players contributed data for individual sessions (*n* = 9 sessions) to ensure robust representations of the actual external loads across the team were gathered. Ethical approval for this study was granted by the Central Queensland University Human Research Ethics Committee (0000024194).

### 2.3 Procedures

#### 2.3.1 Planned external load

The same head coach planned each training session across both seasons by selecting desired drills based on the intended physical, tactical, and technical outcomes sought. Based on historical external load data gathered for each player during specific drills, the coach manipulated the organization of each session to achieve a planned external load target at a team level. These external loads were quantified using the PlayerLoad^TM^ (PL) variable in arbitrary units (AU), with data collected by high-performance staff using microsensor devices (T6; Catapult Sports, Melbourne, Australia). PL is calculated within the manufacturer's software (OpenField, version 2.5.2, build#64421) as the square root of the sum of the squared rate of change in acceleration across all movement planes multiplied by a scaling factor of 0.01 (Fox et al., 2017). The head coach multiplied historical relative PL (AU·min^−1^) values averaged across the entire team by the planned duration (min) to derive a total PL per individual drill. The PL value determined for each drill was summed to give the planned accumulated PL in each session. Planning loads on a drill-by-drill basis (using session-RPE-load) has been shown to yield stronger associations with the actual external loads encountered than planning loads at a sessional level among basketball players (Staunton et al., 2020). The accumulated PL was planned for some drills by selecting from various configurations–for example, factors like team sizes and court space could be altered in game-based drills to elicit different relative PL (O'Grady et al., 2020b; Sosa Marín et al., 2025). The historical external load data were updated every 2 weeks during the pre-season phase and each month during the in-season phase to reflect changes in the external loads encountered for specific drills in each player across time (O'Grady et al., 2020a). The coach reviewed external load data following each session, which could be used in planning subsequent sessions in line with set targets.

#### 2.3.2 Actual external load

Prior to all on-court team training sessions, players were fitted with microsensors secured between the scapulae with vests provided by the manufacturer. Microsensor devices contained an accelerometer, magnetometer, and gyroscope collecting data at 100 Hz, but these data were processed and acquired at 15 Hz (T6; Catapult Sports, Melbourne, Australia). Players wore the same device during all sessions, except when devices failed due to charging issues or hardware faults. In these scenarios, a new device was issued to players to ensure continued data collection. Similar devices (Catapult Vector S7) demonstrate relatively strong inter-unit reliability for PL during court-based team sport activity (intraclass correlation coefficient = 1.00, coefficient of variation = 1.5%; Mackay et al., 2025), supporting the consistency in outputs between different devices.

Microsensor data were captured during each training session for analysis using proprietary software (OpenField, version 2.5.2, build#64421). Data were collected across the entirety of each session with warm-up, cool-down, and non-active periods (i.e., hydration breaks, rest, feedback) between drills excluded from analyses. These periods were identified via live tagging of events by high-performance staff and visual inspection of outputs in the OpenField software. Removal of these periods was necessary given the planned external loads were compiled to reflect participation only during the prescribed drills, not periods outside of the drills. Following each session, data were downloaded from devices, then processed and analyzed via OpenField software. Summary data for each session were then exported and stored in a Microsoft Excel spreadsheet (version 2501; Microsoft Corporation; Redmond, WA, USA). The average accumulated PL (AU) across all players who participated in the session was determined as the actual external load encountered. Moreover, the average relative PL (AU·min^−1^) and duration (min) were taken to help identify whether any discrepancies between planned and actual outcomes were due to misalignments in session intensity and/or duration.

#### 2.3.3 Data processing

Overall, a sample size of 138 training sessions were included in analyses. Monitored sessions were tagged and categorized regarding the key factors analyzed. Firstly, sessions were categorized according to the season in which they were conducted–either “2022–2023” (70 sessions) or “2023–2024” (68 sessions). Secondly, sessions were categorized according to their intended difficulty levels in line with the process adopted by the head coach and high-performance staff. In this regard, sessions with planned accumulated PL < 375 AU were categorized as “low” (38 sessions), sessions with planned accumulated PL of 375–475 AU were categorized as “medium” (70 sessions), and sessions with planned accumulated PL > 475 AU were categorized as “high” (30 sessions). Thirdly, sessions were categorized according to the number of days before a game (with games identified as game day [GD]). A minus sign along with a numeral was used following “GD” to indicate the number of days before upcoming games on which sessions were held. Sessions were categorized from 5 days before games (GD-5) to 1 day before games (GD-1) to align with the typical scheduling of sessions and process to plan external loads adopted by the head coach. Due to the limited number of samples for GD-5 (5 sessions) and GD-4 (11 sessions), data within these categories were combined with those for GD-3 and collapsed into the combined category of GD-3+ (i.e., sessions conducted three or more days from upcoming games; 33 sessions), with categories of GD-2 (23 sessions) and GD-1 (32 sessions) also used. Fourthly, sessions were categorized as occurring in the “pre-season phase” (50 sessions) or “in-season phase” (88 sessions). Given the limited samples obtained, sessions conducted during the post-season phase (3 sessions) were not included in analyses. Finally, sessions were categorized according to the venue of the upcoming game where “home” referred to sessions conducted up to 5 days before games played at home (52 sessions), and “away” referred to sessions conducted up to 5 days before games played at away venues (36 sessions).

### 2.4 Statistical analyses

All statistical analyses were performed using R software (version 4.4.3; R Foundation for Statistical Computing, Vienna, Austria). Variables were first assessed using the Shapiro-Wilk test, which confirmed normality in the data (*p* > 0.05). Consequently, data were descriptively determined as mean ± standard deviation (SD). Because the data were predominantly obtained under paired conditions for the same players, paired *t*-tests–as adopted for similar comparisons in past research (Wilmot et al., 2025; Kraft et al., 2020)–were conducted to identify differences between the planned and actual external loads (separately for accumulated PL, relative PL, and duration), overall across all sessions combined and for each category within each factor (season, session difficulty, proximity to games, season phase, and game venue). Because of the paired data, Cohen's *d*_*z*_ (with 95% confidence intervals [CI]) was calculated–as the mean difference divided by the standard deviation of the paired differences–to quantify the magnitude of difference in each comparison using the *effectsize* R package. Descriptive magnitudes for Cohen's *d*_*z*_ were assigned according to established thresholds (Hopkins et al., 2009), where: *trivial* = *d*_*z*_ < 0.2; *small* = 0.2 ≤ *d*_*z*_ < 0.6; *moderate* = 0.6 ≤ *d*_*z*_ < 1.2; *large* = 1.2 ≤ *d*_*z*_ < 2.0; *very large* = 2.0 ≤ *d*_*z*_ < 4.0; and *extremely large* = *d*_*z*_ ≥ 4.0. All paired *t*-tests were two-sided, and *p* < 0.05 was considered statistically significant. To further assess agreement between planned and actual external loads for each variable, Bland–Altman analyses were conducted separately for each variable using the *ggplot2* R package. For each session, the difference between planned and actual external loads was calculated, with the mean (of both) plotted against the difference. The mean difference (bias) represents systematic deviation, while the 95% limits of agreement (LoA) were calculated as the mean difference ± 1.96 × SD of the differences. Notably, Bland-Altman plots were generated with the mean difference and LoA indicated by horizontal lines and numerical annotations to facilitate interpretation. This approach provides both a visual and quantitative assessment of agreement and allows identification of potential systematic bias or variability across the range of external loads observed. The *dplyr* R package was used for data manipulation across analyses.

## 3 Results

**Figure 1:**
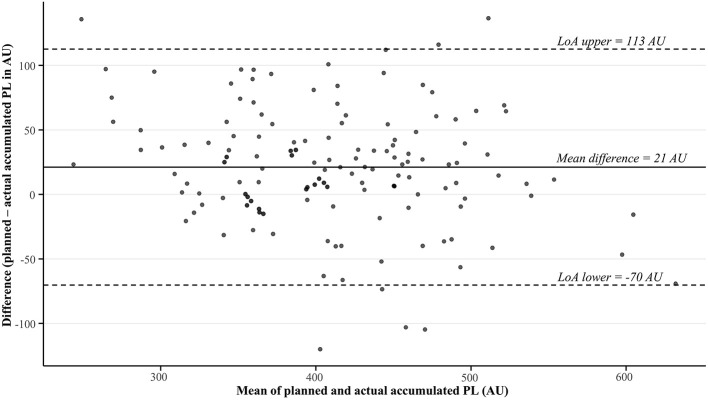
Bland-Altman plot showing the agreement between the planned and actual accumulated PlayerLoad (PL) during on-court team training sessions in a professional, male basketball team across two seasons. AU, arbitrary units; LoA, limits of agreement.

**Figure 2:**
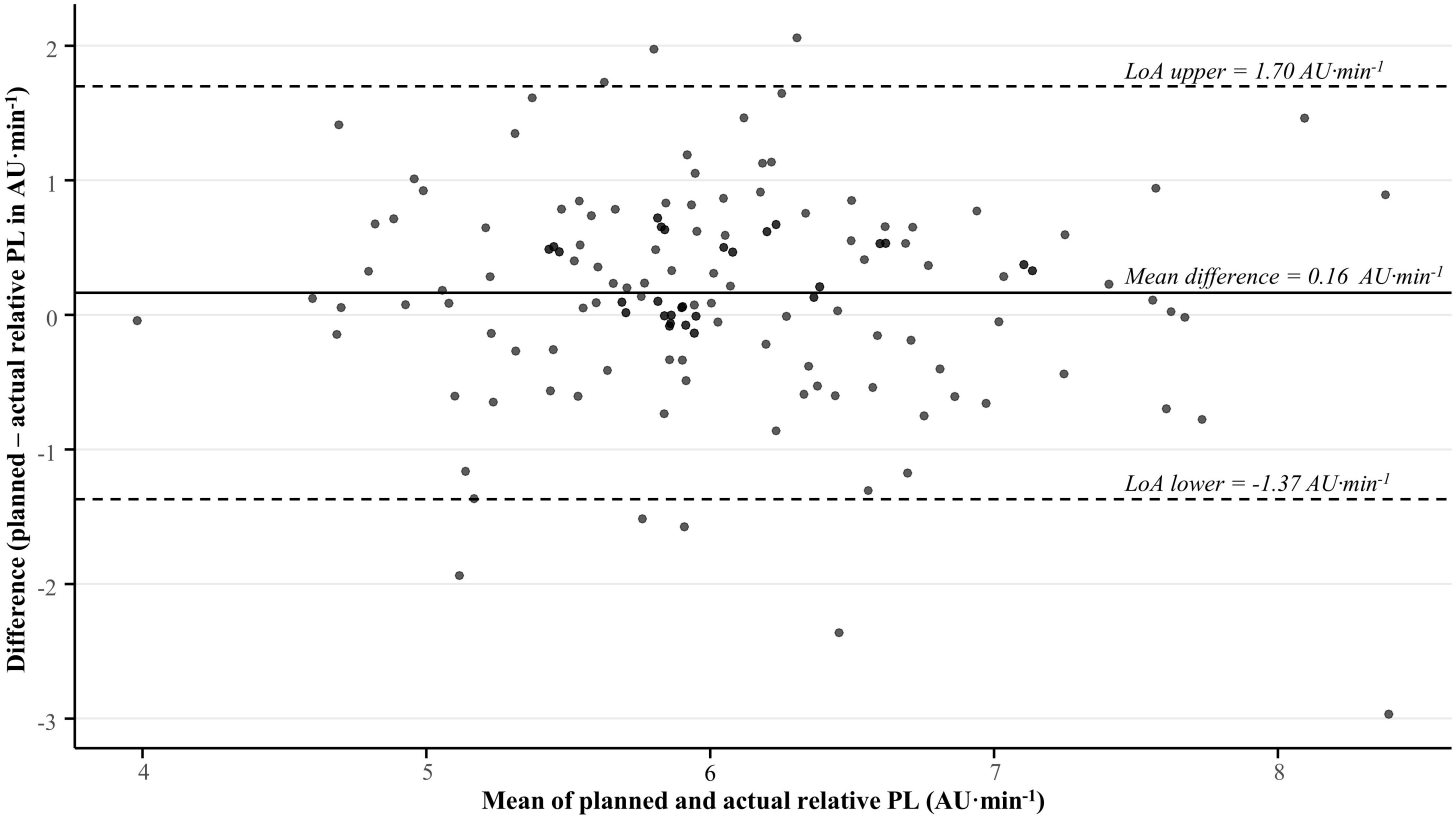
Bland-Altman plot showing the agreement between the planned and actual relative PlayerLoad (PL) during on-court team training sessions in a professional, male basketball team across two seasons. AU, arbitrary units; LoA, limits of agreement.

**Figure 3:**
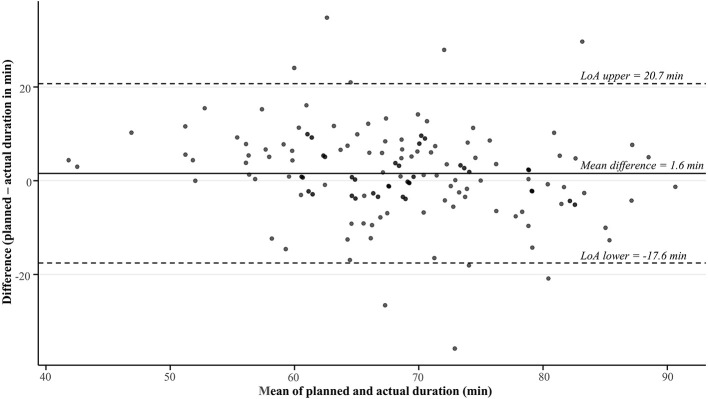
Bland-Altman plot showing the agreement between the planned and actual duration during on-court team training sessions in a professional, male basketball team across two seasons. AU, arbitrary units; LoA, limits of agreement.

The descriptive statistics alongside statistical comparisons regarding the planned and actual external loads–overall and according to each factor–are shown for each variable in Table 1 (accumulated PL), Table 2 (relative PL), and Table 3 (duration). Bland-Altman plots showing the agreement between the overall (not considering any factors) planned and actual external loads are presented separately for each variable in Figure 1 (accumulated PL), Figure 2 (relative PL), and Figure 3 (duration). Analyses revealed the planned accumulated PL was significantly higher than the actual accumulated PL overall, yielding a mean difference of 21 ± 47 AU (Table 1 and Figure 1). This trend was also apparent when considering all categories within each factor (*p* < 0.01), except in the 2022–2023 season, when the intended session difficulty was low, and when sessions were conducted three or more days before games (*p* > 0.05). However, these differences between the planned and actual accumulated PL were predominantly *trivial–small* in magnitude across comparisons, with *moderate* differences only reached in the 2023–2024 season, when the intended session difficulty was high, and when sessions were conducted on the day before games (Table 1).

**Table 1:** Mean ± standard deviation planned and actual accumulated PlayerLoad (PL) during on-court team training sessions overall and according to different factors in a professional, male basketball team across two seasons.

	Accumulated PL (AU)		
Factor			Statistical comparison
	Planned	Actual	
Overall	422 ± 72	401 ± 81	*p* < 0.001^*^, *d_*z*_* = 0.45 [0.28, 0.63], *small*
Season			
2022–2023	421 ± 86	410 ± 100	*p* = 0.11, *d_*z*_* = 0.19 [-0.04, 0.43], *trivial*
2023–2024	424 ± 53	392 ± 55	*p* < 0.001^*^, *d_*z*_* = 0.97 [0.68, 1.25], *moderate*
Session difficulty			
Low	336 ± 26	322 ± 58	*p* = 0.06, *d_*z*_* = 0.31 [-0.02, 0.63], *small*
Medium	426 ± 29	407 ± 53	*p* < 0.001^*^, *d_*z*_* = 0.42 [0.18, 0.67], *small*
High	523 ± 34	489 ± 63	*p* < 0.001^*^, *d_*z*_* = 0.70 [0.29, 1.09], *moderate*
Days before game			
GD-1	340 ± 33	313 ± 54	*p* = 0.002^*^, *d_*z*_* = 0.61 [0.23, 0.98], *moderate*
GD-2	448 ± 54	426 ± 66	*p* = 0.04^*^, *d_*z*_* = 0.45 [0.01, 0.87], *small*
GD-3+	414 ± 55	401 ± 60	*p* = 0.12, *d_*z*_* = 0.28 [−0.07, 0.62], *small*
Season phase			
Pre-season	469 ± 58	447 ± 69	*p* = 0.001^*^, *d_*z*_* = 0.48 [0.18, 0.77], *small*
In-season	396 ± 65	376 ± 77	*p* < 0.001^*^, *d_*z*_* = 0.44 [0.22, 0.66], *small*
Game venue			
Home	389 ± 59	369 ± 66	*p* = 0.006^*^, *d_*z*_* = 0.40 [0.12, 0.68], *small*
Away	407 ± 73	384 ± 90	*p* = 0.006^*^, *d_*z*_* = 0.49 [0.14, 0.83], *small*

^*^indicates a statistically significant difference (*p* < 0.05).

*d*_*z*_ presented alongside 95% confidence intervals in brackets.

AU, arbitrary units; GD, game day.

**Table 2:** Mean ± standard deviation planned and actual relative PlayerLoad (PL) during on-court team training sessions overall and according to different factors in a professional, male basketball team across two seasons.

	Relative PL (AU·min^−1^)		
Factor			Statistical comparison
	Planned	Actual	
Overall	6.13 ± 0.84	5.97 ± 0.91	*p* = 0.02^*^, *d_*z*_* = 0.21 [0.04, 0.38], *small*
Season			
2022–2023	5.94 ± 0.78	5.79 ± 0.98	*p* = 0.16, *d_*z*_* = 0.17 [-0.07, 0.40], *trivial*
2023–2024	6.32 ± 0.86	6.14 ± 0.81	*p* = 0.03^*^, *d_*z*_* = 0.26 [0.02, 0.50], *small*
Difficulty			
Low	5.52 ± 0.73	5.25 ± 0.62	*p* = 0.02^*^, *d_*z*_* = 0.39 [0.06, 0.72], *small*
Medium	6.17 ± 0.76	6.08 ± 0.77	*p* = 0.32, *d_*z*_* = 0.12 [−0.11, 0.36], *trivial*
High	6.80 ± 0.56	6.62 ± 0.95	*p* = 0.25, *d_*z*_* = 0.21 [−0.15, 0.57], *small*
Days before game			
GD-1	5.63 ± 0.77	5.14 ± 0.60	*p* < 0.001^*^, *d_*z*_* = 0.80 [0.39, 1.19], *moderate*
GD-2	6.48 ± 0.68	6.45 ± 0.75	*p* = 0.86, *d_*z*_* = 0.04 [−0.37, 0.45], *trivial*
GD-3+	6.25 ± 1.08	6.32 ± 0.98	*p* = 0.68, *d_*z*_* = −0.07 [−0.41, 0.27], *trivial*
Season phase			
Pre-season	6.21 ± 0.62	6.04 ± 0.76	*p* = 0.09, *d_*z*_* = 0.24 [−0.04, 0.52], *small*
In-season	6.08 ± 0.94	5.92 ± 0.99	*p* = 0.08, *d_*z*_* = 0.19 [−0.02, 0.4], *trivial*
Game venue			
Home	6.12 ± 0.96	5.95 ± 0.93	*p* = 0.12, *d_*z*_* = 0.22 [−0.06, 0.49], *small*
Away	6.02 ± 0.92	5.88 ± 1.08	*p* = 0.35, *d_*z*_* = 0.16 [−0.17, 0.49], *trivial*

^*^indicates a statistically significant difference (*p* < 0.05).

*d*_*z*_ presented alongside 95% confidence intervals in brackets.

AU, arbitrary units; GD, game day.

**Table 3:** Mean ± standard deviation planned and actual duration during on-court team training sessions overall and according to different factors in a professional, male basketball team across two seasons.

	Duration (min)		
Factor			Statistical comparison
	Planned	Actual	
Overall	69.2 ± 9.5	67.7 ± 11.4	*p* = 0.06, *d_*z*_* = 0.16 [-0.01, 0.33], *trivial*
Season			
2022–2023	70.8 ± 10.7	70.7 ± 12.6	*p* = 0.97, *d_*z*_* = 0.01 [−0.23, 0.24], *trivial*
2023–2024	67.6 ± 7.8	64.5 ± 9.1	*p* < 0.001^*^, *d_*z*_* = 0.44 [0.19, 0.69], *small*
Difficulty			
Low	61.5 ± 6.3	61.7 ± 10.4	*p* = 0.92, *d_*z*_* = −0.02 [−0.33, 0.30], *trivial*
Medium	69.9 ± 8.5	67.8 ± 10.5	*p* = 0.05, *d_*z*_* = 0.23 [0.01, 0.47], *small*
High	77.4 ± 7.5	74.8 ± 10.5	*p* = 0.07, *d_*z*_* = 0.35 [−0.02, 0.71], *small*
Days before game			
GD-1	61.0 ± 6.2	61.3 ± 10.1	*p* = 0.90, *d_*z*_* = −0.02 [−0.37, 0.32], *trivial*
GD-2	69.5 ± 7.9	66.8 ± 11.5	*p* = 0.11, *d_*z*_* = 0.35 [−0.08, 0.76], *small*
GD-3+	67.4 ± 9.2	64.4 ± 11.1	*p* = 0.11, *d_*z*_* = 0.29 [−0.06, 0.64], *small*
Season phase			
Pre-season	75.6 ± 7.5	74.3 ± 8.9	*p* = 0.29, *d_*z*_* = 0.15 [−0.13, 0.43], *trivial*
In-season	65.6 ± 8.6	63.9 ± 10.9	*p* = 0.13, *d_*z*_* = 0.17 [−0.05, 0.37], *trivial*
Game venue			
Home	64.0 ± 7.0	62.6 ± 9.0	*p* = 0.27, *d_*z*_* = 0.15 [−0.12, 0.43], *trivial*
Away	67.9 ± 10.2	65.7 ± 13.1	*p* = 0.29, *d_*z*_* = 0.18 [−0.15, 0.51], *trivial*

^*^ indicates a statistically significant difference (*p* < 0.05).

*d*_*z*_ presented alongside 95% confidence intervals in brackets.

GD, game day.

Regarding relative PL, the planned load was significantly higher than the actual load overall (trivial effect), with a mean difference of 0.16 ± 0.78 AU·min^−1^ (Table 2 and Figure 2). This trend was also apparent within some categories for specific factors, including the 2023–2024 season (*trivial* effect), when the intended session difficulty was low (*small* effect), and when sessions were conducted one day before games (*moderate* effect; Table 2). All other comparisons within categories across factors exhibited non-significant (*p* > 0.05), *trivial–small* differences between the planned and actual relative PL (Table 2).

Considering duration, a non-significant, *trivial* difference was apparent between the planned and actual duration overall, with a mean difference of 1.6 ± 9.8 min (Table 3 and Figure 3). Considering categories within each factor, the planned duration was significantly higher than the actual duration only in the 2023–2024 season (*small* effect), with all other comparisons revealing non-significant (*p* >0.05), *trivial*–*small* differences (Table 3).

## 4 Discussion

Our exploratory study showed that the external load (accumulated PL) experienced by the monitored professional, male basketball team was significantly less (*p* < 0.001, *small* effect) than that planned by the head coach during training sessions when considered holistically across two seasons (Table 1). In turn, the lower actual accumulated PL experienced in training sessions predominantly stemmed from a reduced average intensity indicated by relative PL (*p* < 0.05, *small* effect) compared to that planned (Table 2). The observed misalignment between the planned and actual external loads may be attributed to processes adopted both when developing and implementing the training plan.

In developing the planned accumulated PL, the head coach relied upon individualized normative intensities (relative PL) averaged across the team for each drill, which were updated every ~2–4 weeks throughout the season. In this regard, factors like fitness adaptations (Ferioli et al., 2020), fatigue status (Pernigoni et al., 2024), fluctuations in psychological state (Kamarauskas et al., 2022), and player availability could acutely impact the involvement and physical outputs of individual players during training drills, distorting subsequent normative external load intensities when updated across the season. Moreover, the normative relative PL was established for each drill irrespective of the duration over which the drill was performed. In this way, the peak relative PL experienced during basketball training is inversely related to the sample duration (Fox et al., 2021), meaning deviations from planned intensities may be introduced if drills are conducted for longer or shorter periods than customarily performed. Consequently, exploration of other approaches to plan the external load in professional basketball settings are encouraged to counter these issues. For instance, alternative approaches may establish normative relative PL data with more frequent (sessional) updates while considering the duration of specific drills. Technological advancements such as predictive analytics within artificial intelligence systems (Zhou et al., 2025) may assist high-performance staff and coaches in this process by reducing the practical burden involved with data processing while considering complex individualized trends and player combinations across drills within the team.

In implementing the prospectively planned accumulated PL, variations in the actual accumulated PL may have been encountered if the head coach was faced with a different group of players from which the planned relative PL were calculated. In other words, the examined approach involved the head coach planning external loads for each session using normative data from the entire team, which could not account for new players commencing with the team without historical data, and was not updated when individual players missed sessions (e.g., due to injury) or had modified participation prescribed (e.g., rehabilitation plans, added recovery needs). This potential for misalignment is likely further exacerbated by the inter-player variability in PL outputs that can exist within sessions among professional basketball players (López-Sierra et al., 2025). More precisely, players may inherently generate different PL due to varied movement strategies in a given drill (Staunton et al., 2026). So if players who produce considerably larger PL outputs compared to others were to miss sessions, notable reductions in the actual accumulated PL detected across the team would result. Additionally, the head coach may have modified the training contents from that planned in real-time when delivering sessions. Indeed, this notion is supported by survey research (*n* = 44), which indicated that most basketball practitioners modify training at the team (95% of respondents) and player (86% of respondents) levels based on personal observations (Fox et al., 2020). For example, the head coach may have implemented more stoppages for corrections or involved less of the team in some drills to address identified issues directly–both of which reduce the external load intensities of training stimuli (Sosa Marín et al., 2025; Sansone et al., 2023). Consequently, various sources may contribute to misalignments between planned and actual external loads, some of which are likely due to the inherent adaptability required in high-performance training environments (Kinnerk et al., 2023).

In addition to our direct statistical comparisons, Figures 1 (accumulated PL) and 2 (relative PL) visually demonstrate the inconsistency in agreement between the planned and actual external loads across sessions, indicating specific contexts or situations may exacerbate the misalignments observed. In this regard, consideration of contextual factors was a further novel aspect of our analyses, showing misalignment between the planned and actual accumulated PL was most strongly apparent (~7–8%, *p* < 0.001, *moderate* effects) in: (1) the second season monitored; (2) sessions intended to elicit high loads; and (3) sessions held the day before games. First, the lower actual accumulated PL than that planned in the 2023–2024 season may reflect player turnover and performance within the team. For instance, almost half the sample (43%) competed only in the 2023–2024 season. Consequently, new players to the team will require more time to generate historical monitoring data to determine robust normative relative PL values across drills. Likewise, suboptimal performance among the team (winning percentage was 64% in 2022–2023 compared to 43% in 2023–2024) may require greater coaching emphasis on learning than fluency, promoting more instruction and feedback to refine technical and strategic elements (Barry et al., 2025)–which lowers the training intensity compared to that intended (Sosa Marín et al., 2025). Second, the notable discrepancy between the planned and actual accumulated PL in high-load sessions may be related to residual fatigue from prior training and competition within players (Scantlebury et al., 2018), limiting their ability to meet the high outputs desired by the head coach in such scenarios. Alternatively, players may have paced themselves within the wider group setting when sessions became more demanding and they were faced with strenuous tasks (Brink et al., 2014). Indeed, the non-significant reduction in the actual relative PL (Table 2) compared to that planned in these high-load sessions partially supports the notion that players did not reach the intensities that were intended. Moreover, the non-significant reduction in actual duration (Table 3) suggests that subtle misalignment in session timings were also apparent to further contribute to variations between the planned and actual PL in high-load sessions. Third, the greater mismatch between the planned and actual accumulated PL on the day before games within weekly microcycles likely reflects a conservative approach from the head coach in tapering their players leading into competition (Mujika et al., 2018). In this regard, basketball coaches typically structure lower loads immediately before games to optimize player readiness (Iglesias et al., 2024; Wellm et al., 2023; Salazar et al., 2025), with additional adjustments potentially needed to further “freshen” some players within the team.

Comparisons between our findings and those made previously are difficult, given that most research on this topic in basketball (Kraft et al., 2020; Staunton et al., 2020) and wider sports (Paul et al., 2021) has compared planned and actual training loads using perceptually-based, internal RPE metrics. Nevertheless, these previous findings somewhat align with our results, given the RPE reported by male and female, collegiate basketball players were significantly lower (~14–29%, *p* < 0.05) than those planned by their head coaches (Kraft et al., 2020). In wider team sports, and considering session difficulty, most male, national-level volleyball players (63%) identified training sessions that were intended to be hard by the head coach (RPE = >5) as easy (RPE = < 3) or moderate (RPE = 3–5; de Andrade et al., 2014). Similarly, school-based adolescent athletes (17.4 ± 0.8 years) from various team sports reported notably lower RPE (~13%) than those planned by head coaches only when sessions were intended to be hard (RPE = 5.2 ± 0.6; Scantlebury et al., 2018). In contrast, national-level, young soccer players (under-19 years and under-17 years) reported significantly higher RPE and session-RPE loads (~2–3%, *p* < 0.001) in training than those prescribed by their coaches (Brink et al., 2014). However, within this study–and similar to what we observed–comparable (*p* > 0.05) planned and actual session durations were apparent, and sessions prescribed to be hard (RPE = >14) were perceived as significantly less demanding (~6%, *p* < 0.001) than planned by coaches (Brink et al., 2014). In the only other study contrasting planned and actual training loads using external load metrics, a lack of agreement was reported with female, professional soccer players covering more total distance (5,162 ± 1,223 m vs. 4,956 ± 1,134 m) and less high-intensity distance (63 ± 44 m vs. 294 ± 168 m) than planned by their coaching staff (Wilmot et al., 2025). Consequently, our research contributes importantly to the existing evidence base in this area, which collectively suggests athletes may complete lower training loads than those planned by their coaches, particularly when considering intensity-based metrics and during sessions intended to be hard.

Despite providing novel findings that are practically informative for end-users, the prominent limitations we encountered should be considered. First, we examined a specific systematic approach in planning the external load adopted within the monitored team without any modification. Consequently, key constraints within this approach centered on establishing normative load data by: (1) using PL as the key metric; (2) updating values for each drill across the team every ~2–4 weeks; (3) incorporating the whole team irrespective of which players actually participated in each session; and (4) determining consistent intensities for each drill regardless of their duration. In particular, variations in player availability and PL outputs may have impacted determination of normative relative PL used in planning sessions as well as the actual accumulated PL detected in sessions, which were predicated on team-based averages. Second, the planned and actual external load were compared at the sessional level. Further analyses could extend on our work by concomitantly exploring this topic at sessional and drill levels to identify specific tasks or scenarios that may yield the greatest misalignment between planned and actual external loads. In support of this notion, the accuracy in anticipating the relative PL from normative data established from training sessions has been shown to vary across different 5v5 games-based drills among male, semi-professional basketball players (O'Grady et al., 2020a). Third, we did not record any data from the head coach pertaining to their decision-making processes when implementing the training plan–future research on this topic is therefore encouraged to incorporate such qualitative assessment. Fourth, our study was restricted to a single team–under the guidance of a single head coach who planned the training loads–so the outcomes may not be indicative of those derived from other teams within the NBL or wider competitions. Indeed, practical constraints (e.g., access to hardware and software, expertise, labor, and coach preferences) may prohibit the implementation of such systems to plan and monitor the external load in some teams, particularly those at non-professional levels (Fox et al., 2020; Timmerman et al., 2024). Moreover, recruiting a single team limited our ability to explore some factors more precisely (e.g., splitting the in-season into phases, examining all days within the weekly microcycle) and conduct further analyses according to playing position and role (Shirley et al., 2026). Finally, we used an arbitrary cut-point of participation for at least 80% of individual sessions for player data to be included in analyses. This cut-point was chosen to ensure actual external load data were representative of a substantial portion of the session (given the head coach planned for participation in the entire session) alongside maximizing the sample size (138 sessions). In support of this approach, comparable outcomes were obtained in preliminary analyses we conducted using a subset of data across 40 sessions involving 100% participation among the included players.

## 5 Practical applications

The lower than intended accumulated and relative PL we observed among players, suggests they may have received insufficient stimuli to promote the desired adaptations sought by the head coach (Paul et al., 2021). However, given training effects are largely driven by the internal load (e.g., cognitive, cardiovascular, neuromuscular, and metabolic demands; Mercer et al., 2022), further analyses incorporating such measures are warranted to confirm the effects of such misalignments on player adaptive responses. In turn, while we observed significant effects, it is important to note that the overall misalignment between the planned and actual accumulated PL (~5%) and relative PL (~2%) were *small* in magnitude (Tables 1 and 2), but with rather wide variation (Figures 1 and 2). Likewise, the relatively comparable (~2%, *p* > 0.05, *trivial* effect) planned and actual session duration (Table 3 and Figure 3) evident across all sessions combined suggests that the head coach implemented session timings in a controlled manner, consistent with those intended. The level of agreement between the planned and actual external load deemed to be acceptable likely depends on end-users' preferences and standards, which will vary across settings. Moreover, there will always likely be some misalignment in such systems, given the strong reliance on coach observation to identify key performance areas (e.g., psychological indicators, movement skills, and game intelligence; Rogers et al., 2022) and modify training contents during sessions (Fox et al., 2020) among basketball teams. In this way, coaches will plan the training contents–including the intended external load–leading into sessions, but they will likely use their expertise to adjust training plans in real-time by deciding when to push (or not push) players, when to deliver instructional or evaluative feedback, and when to interact with players (Timmerman et al., 2024). Consequently, these factors should be considered when evaluating the level of agreement observed between planned and actual external loads in team sport environments.

Considering the limitations we identified, future work should extend upon the system we explored to further strengthen the agreement between the planned and actual external loads in basketball and research practice. Consequently, we have provided some key recommendations to consider when planning and implementing intended external loads in basketball settings in Table 4.

**Table 4:** Research and practical recommendations to consider when planning and implementing intended external training loads in basketball.

Recommendation	Reasoning	Further considerations
Incorporate wider metrics than PlayerLoad	More specific metrics (e.g., vertical, accelerative, and change-of-direction indices) or those collected with other technologies (e.g., local positioning systems) might provide useful additional insight (Torres-Ronda et al., 2022; Burger et al., 2024) Internal load metrics will enable a better understanding of potential adaptive implications in players (Mercer et al., 2022)	Added burden to collect, process, interpret, and communicate data (West et al., 2021) Potential to explore application of artificial intelligence systems (Zhou et al., 2025)
Update normative data frequently	Updating data more frequently (e.g., each session) (West et al., 2021) will better negate the factors promoting acute changes in the actual external training load, like fluctuations in fitness and fatigue (Ferioli et al., 2020)	Regularity of updates will depend on the time (e.g., training schedule) and resources (e.g., staff) available
Consider only players who are going participate	Developing planned external training loads incorporating only those likely to participate will enhance specificity	Late changes to player availability (e.g., illness) or involvement (e.g., added recovery needs) will hinder this process
Establish normative values for drills dependent on their duration	Given exercise intensity is a function of duration, drill intensities will likely decrease the longer they are performed (Sansone et al., 2019)	A greater scope of normative data for each drill will need to be determined by staff and considered by coaches
Collect relevant data when implementing planned sessions	Detecting changes (and reasoning for them) to the planned training contents within sessions will strengthen understanding of any observed misalignments and better inform any needed actions	Qualitative data from the coach will depend on their availability and willingness to contribute in this way (Fullagar et al., 2019; Timmerman et al., 2024)
Consider factors of interest in analyses	Identifying problematic factors or scenarios where misalignments occur could inform development of improved player preparation strategies Analyses may also be conducted at the individual player level for greater specificity (Timmerman et al., 2024)	Identified factors must be relevant with practical utility for the team context Artificial intelligence systems might be useful in recognizing complex patterns in data to help identify key factors (Zhou et al., 2025)

## 6 Conclusions

Our exploratory study adds to the limited body of evidence contrasting planned and actual external loads during training in the wider team sport literature (Wilmot et al., 2025). Adopting a retrospective design exploring data acquired in a real-world, professional basketball setting strengthened the ecological validity of the outcomes we obtained. In this way, the significantly lower accumulated PL encountered was primarily attributed to a reduced session intensity (relative PL) compared to that planned by the head coach. This misalignment was particularly apparent in the second season monitored, in sessions prescribed to deliver high accumulated PL, and in sessions conducted on the day before games. In turn, comparable planned and actual session durations indicate that the head coach controlled session timings as intended. Our findings highlight the extent of misalignment–which were *trivial*–*small* in magnitude–between planned and actual external loads using a specific system adopted in the monitored team. Accordingly, end-users may deem this level of agreement acceptable if it fits their personal preferences and standards, or alternatively, end-users may follow the provided recommendations where practicable to enhance this level of agreement.

## Data Availability

The raw data supporting the conclusions of this article will be made available by the authors, without undue reservation.
